# Gemination of an Erupted Mandibular Third Molar: A Short Presentation of an Exceptionally Rare Clinical Occurrence

**DOI:** 10.1055/s-0043-1772248

**Published:** 2023-09-20

**Authors:** Hans Ulrich Brauer, Andreas Bartols

**Affiliations:** 1 Dental Academy for Continuing Professional Development, Karlsruhe, Germany; 2 Clinic for Conservative Dentistry and Periodontology, Christian-Albrechts-University Kiel, Germany

**Keywords:** double tooth, fusion, gemination, third molar, geminated tooth of 48, dental anomalies

## Abstract

Double teeth, like fusions and geminations, are rare disorders of tooth development. In this short case presentation, we describe the unique appearance of a fully erupted mandibular wisdom tooth in a 72-year-old patient whose tooth exhibited gemination. This was possible because tooth 46 had to be removed from the patient at the age of 20 and the missing molar was not replaced. This geminated tooth of 48 had been in function for almost 50 years and finally had to be removed due to a periodontal inflammation. To the best of our knowledge, this is the first case presented of this dental anomaly for a fully erupted lower wisdom tooth.

## Introduction

Both in the first dentition and in the permanent dentition, numerous tooth development alterations are possible, which lead to a special tooth shape. Different terms are used to describe such dental twin anomalies, especially concrescence, fusion, gemination, and double teeth. 1 2 Occasionally, there is some confusion regarding the correct use of these terms, and an exact diagnosis is not always possible clinically. 3 4 5 6

Concrescence as dental twin anomaly consists only of a union between two or more teeth over the cementum in either deciduous or permanent dentition. 1 6 7 This means that only the roots of two adjacent teeth are fused. Concrescence is often found in maxillary molars. 7 Concrescence can be further divided into an acquired and true form with regard to its possible etiology. The true concrescence occurs between two developing teeth, whereas the acquired form develops between two fully formed teeth. 7

Fusion (also referred to synodontia or false gemination) is defined as a union between the dentin of two or more separately developing teeth. The fusion may be total or partial. Consequently, the fusion leads to a reduced number of teeth in the dental arch. 2 For clinical differentiation between fusion and gemination, the Mader's two teeth rule can be used. 8 The etiology of this anomaly is still unknown. A discussed cause is the close contact and pressure between two germs that can result in fusion. 2 9 The most commonly affected teeth are incisors in the upper jaw. 2

Gemination is defined as a single enlarged or joined (duplicated) tooth, with the number of teeth present being normal if the anomalous tooth is counted as one. 2 Gemination results in a developmental aberration of ectoderm and the mesoderm. According to Pindborg, gemination is thought to be due to an incomplete attempt of one germ to divide into two teeth. 10 The resulting tooth has two crowns or a large partially separated crown. 5 Clinically, gemination is indicated by a groove or depression in the crown of the tooth that delineates the two teeth. 9 This failed attempt may result in two crowns of the same size with normal dimensions, or one of the crowns is rudimentary. 9

Many theories have been proposed for the etiology of gemination, especially environmental factors, dental traumas, vitamin deficiencies, systemic diseases, and genetic predispositions. 11 The prevalence for fusion and gemination is low and mostly reported in the literature as 0.05 to 1%. A recent study of 3,000 patients identified only one case (0.03%) of geminated teeth and two cases (0.06%) of fusions. 12 The permanent dentition seems to be affected more frequently. Bilateral forms are less common than unilateral forms. 13 14 Gender does not seem to play a role. 12 Geminations cannot be assigned to an ethnicity or population group.

Treatment of double teeth in the anterior region may be required for orthodontic or esthetic reasons. 2 Irregular shapes in the mesiodistal dimension of roots and/or crowns, often associated with crowding of the anterior teeth, frequently require orthodontic treatment including extraction of the malformed tooth. 2 No uniform therapy concepts exist in the literature for the posterior region due to the low prevalence and patients are usually treated on an individual case basis. 15 In single cases, cone-beam computed tomography (CBCT) is used for the diagnosis of double teeth and treatment planning. 4 5 6 9 15 16 For example, Buchanan et al concluded in a case of a 12-year-old girl with a double gemination that the use of three-dimensional (3D) imaging greatly aids treatment planning and communication with the patient in such uncommon and unusual cases. 4

Double teeth are not an exact dental diagnosis, but are often used as a catch-all term when the diagnosis is unclear. Precisely because the distinction between fusion and gemination is often difficult, some authors recommend using the neutral term “double teeth” in these cases. Fused and geminated teeth are reported more common in anterior maxillary teeth. Reports on geminations in molars are a rarity and mostly concern the maxilla. There are only a few case reports of geminated teeth in the mandibular posterior region, involving either first or second molars 4 5 or cases with displaced wisdom teeth that have not erupted. 11 17 18

Here, we present a unique case with gemination of a fully erupted, lower third molar. The tooth found its way into the tooth row and was in function for almost five decades. According to the best of our knowledge, this is the first case presented of this dental anomaly for a fully erupted lower wisdom tooth.

## Case Presentation

**Fig. 1:**
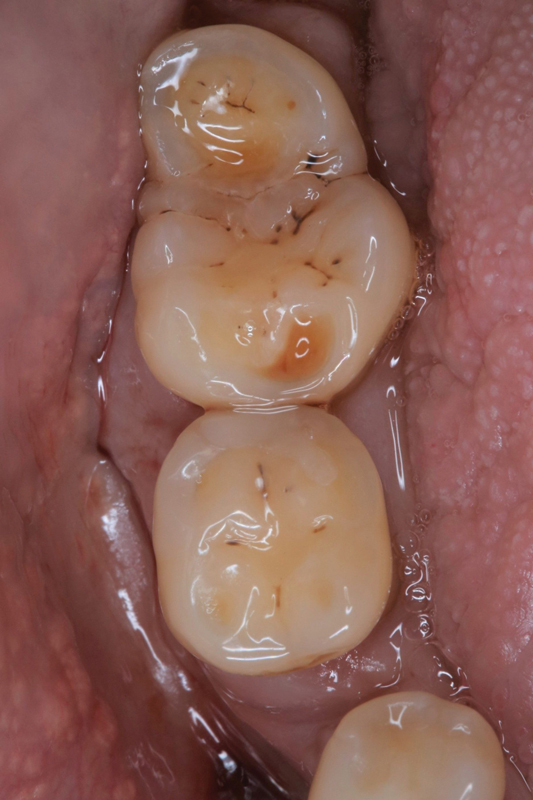
Clinical image of the tooth structure in region 48. The tooth 46 is missing. The suspected diagnosis of geminated tooth of 48 was made.

A 72-year-old patient presented to our outpatient clinic complaining of pain and discrete intraoral swelling in the right posterior region of the mandible for several days. There was no history of dental or orofacial trauma. Clinical examination revealed that tooth 46 was missing and teeth 47 and 48 were present. In the right wisdom tooth region, the third molar appeared as a double tooth, and initially it was clinically unclear whether it was a fusion or a gemination ( Fig. 1 ).

**Fig. 2:**
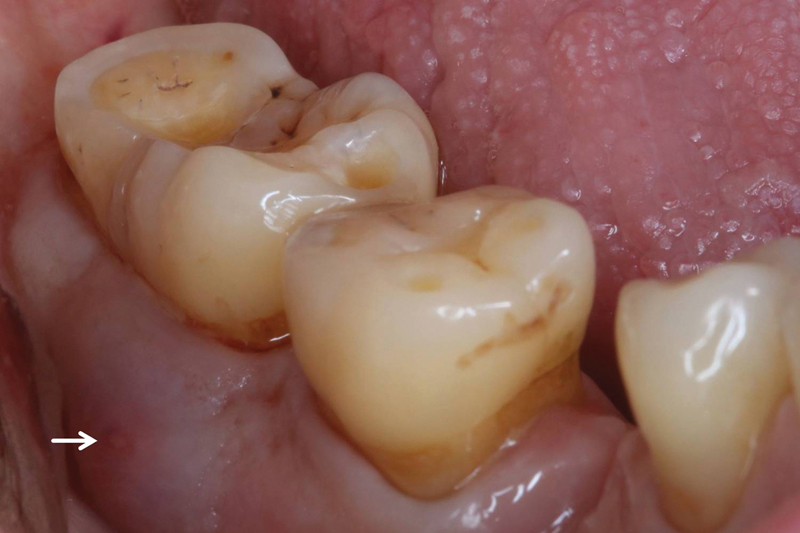
Anterior view: swelling and fistula (white arrow) in the buccal mucosa.

The patient reported that the tooth structure had always been present and that it had not caused any problems so far. Clinical inspection further revealed that the pocket probing depth was significantly increased at 10 mm and that a fistula was present in the buccal mucosa in the wisdom tooth region ( Fig. 2 ).

**Fig. 3:**
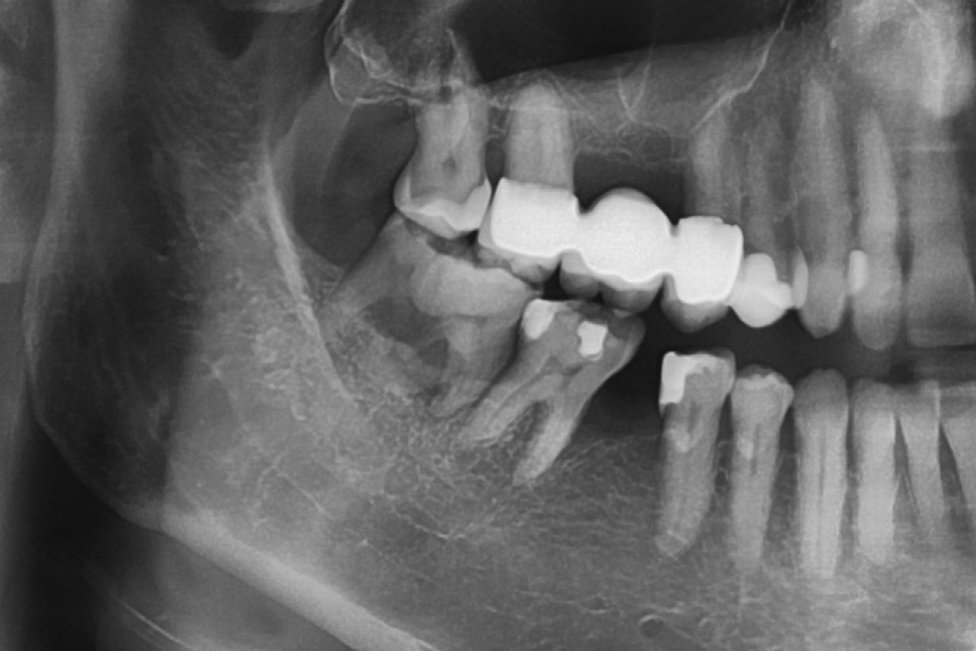
Detail of the panoramic radiograph showing the geminated third molar 48. The tooth 46 is missing.

**Fig. 4:**
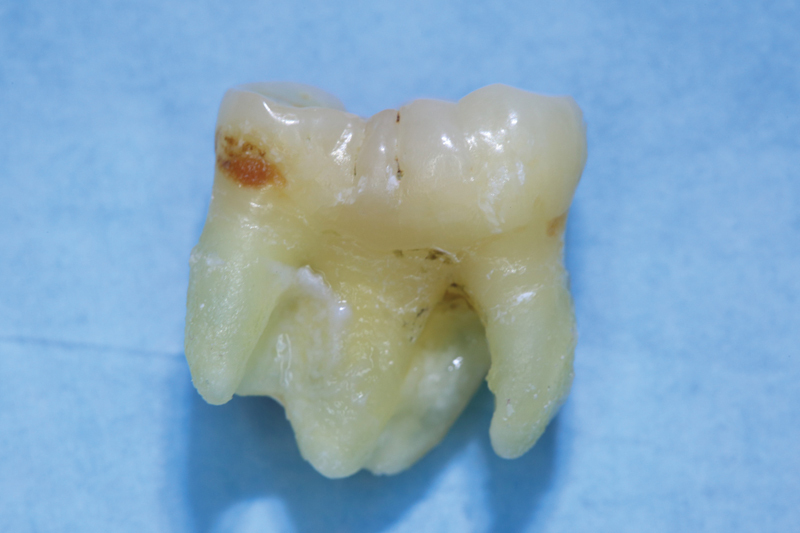
Buccal view of the geminated tooth of 48.

**Fig. 5:**
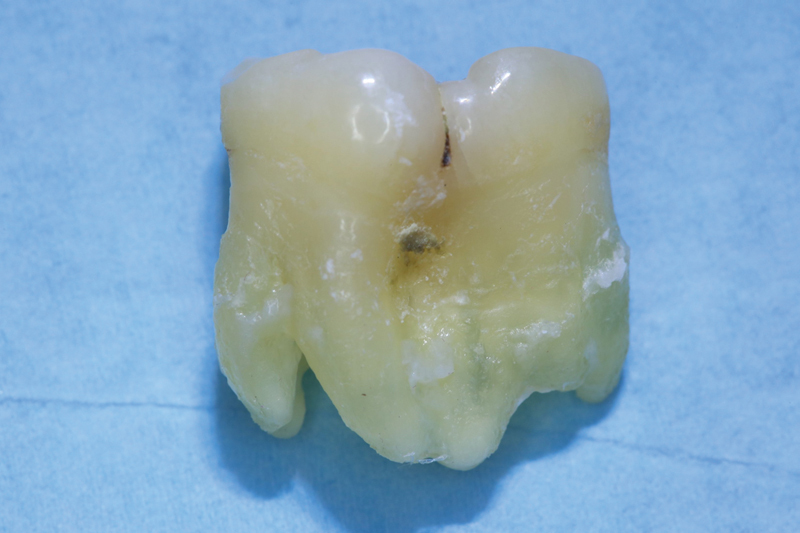
Lingual view of the geminated tooth of 48. The large crown is partially separated.

The sensitivity test performed with CO2 snow was positive. According to the Miller classification, the tooth had a grade 1 degree of looseness. By applying gentle pressure to the area of intraoral swelling, exudate could be expressed through the fistula opening. A panoramic radiograph was then taken ( Fig. 3 ). On the radiograph, the large radiopaque mass was found in the mandibular right third molar region. The radiological appearance was highly suggestive of gemination of the third molar during development and therefore a geminated tooth of 48 was diagnosed. Due to advanced vertical bone resorption and increased pocket probing depths with the presence of a fistula, the tooth had to be removed. Here, CBCT was not used for radiation exposure reasons and was not indicated from a surgical point of view. According to the patient, there also was no 3D imaging of the head and neck region in the past. The tooth removal was uneventful and the diagnosis of gemination was confirmed at surgery ( Figs. 4 and 5 ).

The original treatment plan was to place two endosseous implants in region 46 and 47 with two single-tooth crowns as a superstructure. Due to the patient's general medical situation that occurred in the meantime, the patient decided against a prosthetic restoration in the IV quadrant and consciously opted for the shortened dental arch concept. The patient was informed about a possible elongation of wisdom tooth 18.

**Fig. 6:**
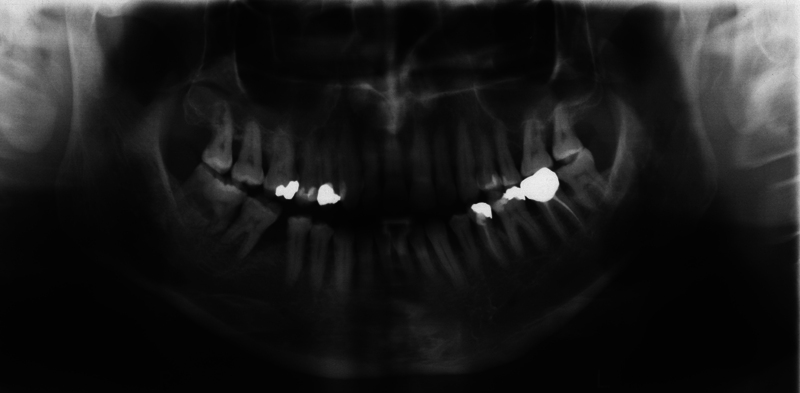
Panoramic radiograph taken 20 years prior to tooth removal shows the geminated tooth of 48. Tooth 46 was already missing at that time. Third molar 38 can be described as normal in size and shape.

When specifically addressed, the patient recalls that the lower right first molar had to be removed at around the age of 20 due to recurrent pain. However, the exact dental diagnosis remains unknown. Whether gemination was already present at that time is not remembered by the patient. The patient had no orthodontic treatment. During routine dental checkups, however, he had been repeatedly asked about his particular tooth over the past decades. For him, it had always been just a normal tooth. After appropriate research, a conventional panoramic radiograph taken 20 years before the tooth removal could be organized. Even then, the radiography shows the missing mandibular tooth 46 and the geminated tooth of 48. On the opposite side, third molar 38 is normal ( Fig. 6 ).

**Fig. 7:**
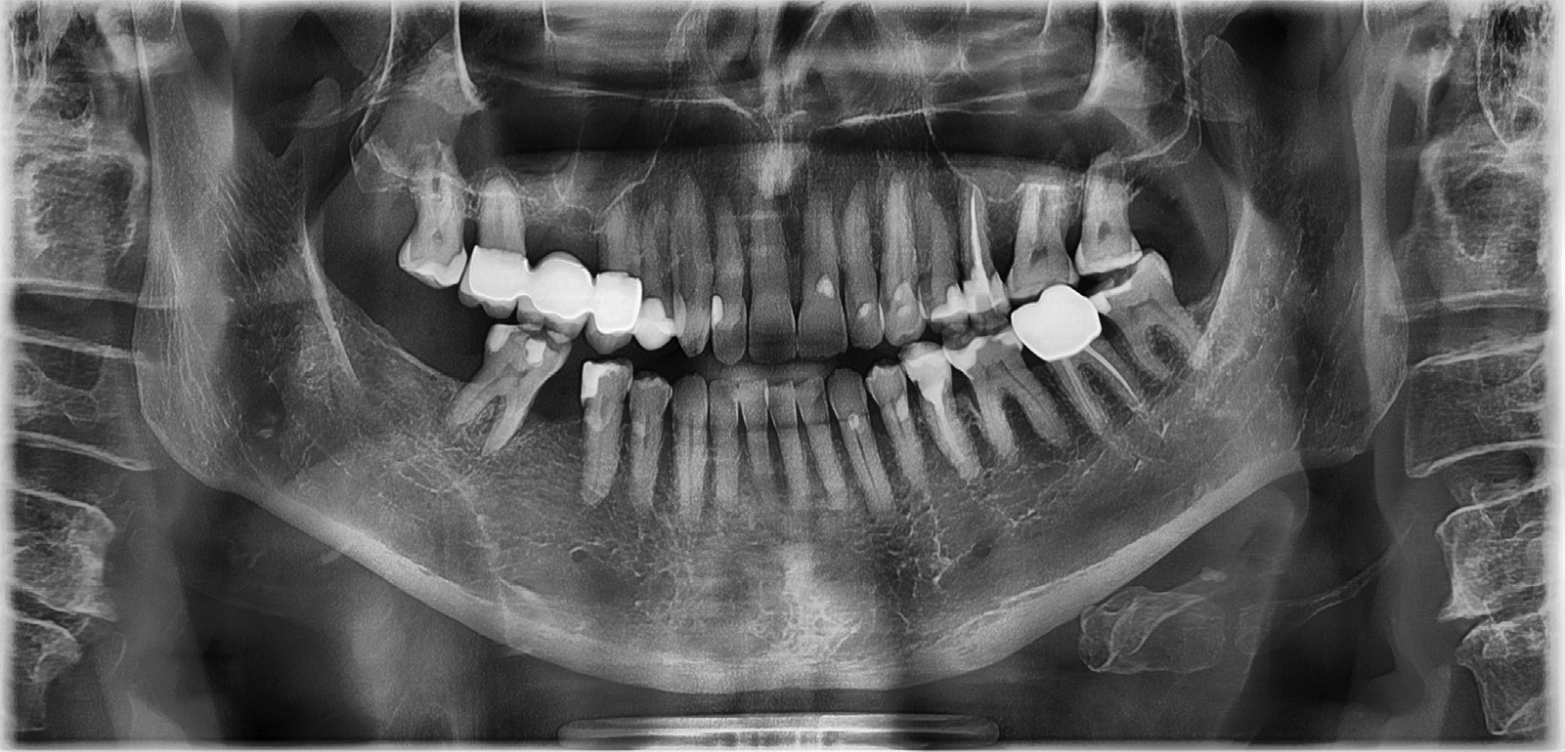
The panoramic scan 2 years after the removal of the gemination is completely ossified in region 48. Tooth 18 appears only slightly elongated. The position of tooth 47 has not changed.

A control radiograph 2 years after tooth removal shows normal bony conditions ( Fig. 7 ).

## Discussion

Gemination or fusion is a rare occurrence in the mandibular posterior teeth. 15 There are only a few reports of geminated lower molars and third molars in the literature. Sandeep et al reported a case of a 30-year-old female with complaint of pain in her right lower back tooth region for the last 1 month. The panoramic radiograph showed a partially erupted atypical tooth that appeared to have two crowns. A radiological diagnosis of wisdom tooth with fused supernumerary teeth was made. Surgical removal was performed under local anesthesia. The examination of the extracted third molar revealed the continuation of the tooth except they appeared separated by a marked groove on the buccal and lingual surface. The molar was having a single root and thereby looked like an incomplete division of the tooth. Therefore, the diagnosis of gemination was made. 19 Hernandez-Guisado et al. reported a case of a 19-year-old male with the appearance of episodes of pericoronitis of the left inferior third molar. In this case, surgical extraction of the tooth was performed. The extracted tooth had double dimensions and a deep furrow is seen as well in the place where the union of both germs took place. The diagnosis geminated third molar was made. 11 In another case report of a geminated lower posterior tooth 47 in an 11-year-old male, no therapy was chosen. 5

Double teeth in the posterior region usually do not have enough space to erupt and adjust in the dental arch due to their enlarged crown. If third molars with such infrequent anomalies are discovered in the teenage years on a panoramic radiograph, surgical removal of these teeth is usually recommended. 11 17 19 The reason is that these double teeth can potentially cause complications with surgical removal at a later time or can also cause problems with the continued retention of these dental anomalies. These problems include especially caries and crowding. In our presented case, tooth 46 was removed from the patient at the age of 20 due to pain. At that time, the technology of CBCT was not yet available for a radiographic examination of this tooth anomaly in region 48. Of course, conventional CT could have been used for clarification. The result of this special case constellation was that the geminated tooth was able to erupt and also fit into the tooth row without orthodontic therapy. According to the patient history, this tooth had been in function for almost 50 years. When the double tooth erupted ultimately remains unclear. It must be assumed that this took place a few years after the removal of tooth 46 and mesialization of tooth 47. The double tooth shows the characteristic features of gemination clinically and radiographically. The tooth crown is enlarged, resembles two teeth, and has a retraction as separation of the enlarged tooth crown. The rootstock consists of several roots with a contiguous pulp. Another special feature to be noted is that the removed tooth has no caries. From the authors' point of view, an attempt to preserve the double tooth in the case presented was not seriously considered. Theoretically, one could have tried to treat the tooth endodontically in order to subsequently attempt a hemisection of the tooth and thus a partial preservation of the tooth. However, this would have been a procedure with a very uncertain prognosis.

The authors agree with Hernandez-Guisado et al. that the distinction between fused and geminated third molars is uncertain, because we cannot be sure whether the supernumerary tooth originates from the germ or the later fused tooth lamella. 11 In these special cases, the use of the term double teeth has its justification. The general dentist should be familiar with the common dental anomalies. The present case shows that it is quite possible for teeth and especially wisdom teeth with rare anomalies to prove themselves clinically in the patient's mouth over many years and do not always have to be removed reflexively and inevitably, but this must be considered on an individual basis.

## Conclusion

The clinical diversity of tooth development disorders is challenging. The case impressively shows that in the case of special constellations a geminated mandibular third molar can erupt into the oral cavity and remain there in function for decades. Even in the case of asymptomatic, displaced wisdom teeth with gemination, careful consideration must always be given to whether it is possible to leave them in place and even integrate them into the tooth row, due to the complications that can potentially occur if they are removed.
